# Characterisation and Carriage Ratio of *Clostridium difficile* Strains Isolated from a Community-Dwelling Elderly Population in the United Kingdom

**DOI:** 10.1371/journal.pone.0022804

**Published:** 2011-08-23

**Authors:** Fabio Miyajima, Paul Roberts, Andrew Swale, Valerie Price, Maureen Jones, Michael Horan, Nicholas Beeching, Jonathan Brazier, Christopher Parry, Neil Pendleton, Munir Pirmohamed

**Affiliations:** 1 National Institute for Health Research (NIHR) Biomedical Research Centre, Royal Liverpool and Broadgreen University Hospitals National Health Service (NHS) Trust, Liverpool, United Kingdom; 2 Department of Pharmacology, The Wolfson Centre for Personalised Medicine, University of Liverpool, Liverpool, United Kingdom; 3 Directorate of Infection and Immunity, Royal Liverpool and Broadgreen University Hospitals NHS Trust, Liverpool, United Kingdom; 4 Neurodegeneration Research Group, School of Community-Based Medicine, University of Manchester, Manchester, United Kingdom; 5 Clinical Group, Liverpool School of Tropical Medicine, University of Liverpool, Liverpool, United Kingdom; 6 Anaerobe Reference Unit, Public Health Laboratory, University Hospital Wales, Cardiff, United Kingdom; 7 Division of Clinical Infection, Microbiology and Immunology, Institute of Global Health, University of Liverpool, Liverpool, United Kingdom; 8 Wellcome Trust Major Overseas Programme, Mahidol-Oxford Tropical Medicine Research Unit, Bangkok, Thailand; Charité, Campus Benjamin Franklin, Germany

## Abstract

**Background:**

Community-associated *Clostridium difficile* infection (CDI) appears to be an increasing problem. Reported carriage rates by *C.difficile* are debatable with suggestions that primary asymptomatic carriage is associated with decreased risk of subsequent diarrhoea. However, knowledge of potential reservoirs and intestinal carriage rates in the community, particularly in the elderly, the most susceptible group, is limited. We have determined the presence of *C.difficile* in the faeces of a healthy elderly cohort living outside of long-term care facilities (LCFs) in the United Kingdom.

**Methods:**

Faecal samples from 149 community-based healthy elderly volunteers (median age 81 years) were screened for *C.difficile* using direct (Brazier's CCEY) and enrichment (Cooked Meat broth) culture methods and a glutamate dehydrogenase (GDH) immunoassay. Isolates were PCR-ribotyped and analysed for toxin production and the presence of toxin genes.

**Results:**

Of 149 faecal samples submitted, six (4%) were found to contain *C.difficile*. One particular sample was positive by both the GDH immunoassay and direct culture, and concurrently produced two distinct strain types: one toxigenic and the other non-toxigenic. The other five samples were only positive by enrichment culture method. Overall, four *C.difficile* isolates were non-toxigenic (PCR-ribotypes 009, 026 (n = 2) and 039), while three were toxigenic (PCR-ribotypes 003, 005 and 106). All individuals who had a positive culture were symptom-free and none of them had a history of CDI and/or antibiotics use in the 3 month period preceding recruitment.

**Conclusions:**

To our knowledge, this is the first study of the presence of *C.difficile* in healthy elderly community-dwelling individuals residing outside of LCFs. The observed carriage rate is lower than that reported for individuals in LCFs and interestingly no individual carried the common epidemic strain PCR-ribotype 027 (NAP1/BI). Further follow-up of asymptomatic carriers in the community, is required to evaluate host susceptibility to CDI and identify dynamic changes in the host and microbial environment that are associated with pathogenicity.

## Introduction

In the last decade, there has been an upsurge in the prevalence and severity of *Clostridium difficile* infection (CDI) such that it has become a major healthcare problem [1]. This has coincided with the emergence of more virulent epidemic strains, particularly PCR-ribotype 027 (NAP1/BI) that have caused hospital outbreaks in North America and Europe with high morbidity and mortality [2]. There is growing evidence for the existence of significant functional differences between epidemic and non-epidemic historical types from isolates of the same ribotype/toxinotype group [3], [4], possibly associated with a strong selective pressure due to broad antimicrobial usage. Community-associated CDI appears to be adding to this disease burden [5], although this may have been underreported.

Relatively little is known about potential community reservoirs and intestinal carriage rates of *C. difficile* in individuals living outside health care facilities. In neonates, for example, the carriage frequency is significantly higher than in any adult group [6], [7]. Although neonates rarely develop CDI, they may act as important reservoirs and there is evidence of cross-colonisation amongst children in day-care facilities, nurseries and kindergartens [7]. Potential sources of *C. difficile* in the hospital setting include both symptomatic and asymptomatic patients and contaminated environments [8]. Riggs *et al.* [9] suggested that asymptomatic patients contribute to CDI in long-term care facilities (LCFs), but it is uncertain whether intestinal carriage by healthy adults in the community acts as a reservoir and a potential source for community-associated CDI. Conversely, some authors suggested that asymptomatic carriage of *C. difficile* was associated with decreased risk of subsequent diarrhoea [10], while others believed that *C. difficile* was even not part of the normal faecal flora in elderly in-patients [11].

Carriage and transmission of *C. difficile* has been studied in the elderly living in LCFs [12], [13], as well as in healthy adults aged up to 65 years (median age 22 years) in Japan [14], [15], many of whom were University students. Reported asymptomatic carriage rates of the organism are contentious and vary up to 5-fold across several studies (4–20%) [9], [12]–[20], in part due to differences in the population target, such as age, health and dwelling status, as well as the adoption of different culture and detection standards. To the best of our knowledge, there are no studies of carriage in healthy elderly individuals aged 65 years and over residing outside of LCFs. We have therefore studied the prevalence of asymptomatic faecal carriage of *C. difficile* in a healthy elderly cohort in the United Kingdom. Isolates were investigated to determine whether the strains isolated from these individuals were current endemic PCR-ribotypes and to assess their characteristics and distribution.

## Materials and Methods

Ethics Statement: Ethical approval was obtained from the Liverpool Research Ethics Committee (ref. 08/H1005/32), and each participant volunteer gave written informed consent.

Two hundred and six members of a large, well studied longitudinal cohort of healthy elderly volunteers [21] residing in their own homes in different geographical areas in the north of England, mostly from Manchester and Newcastle metropolitan areas [Supporting Information – Figure S1], were approached and initially agreed to take part of a prospective study of CDI. All individuals were of white Caucasian origin and were enrolled as healthy age-matched controls with no clinical evidence of CDI.

A sample collection package was sent to participating volunteers with accompanying instructions. The package also contained a questionnaire which included questions about age, gender, a history of CDI in the preceding year and the details of any medication taken. Details of acid suppressive agents, including proton pump inhibitors, and antimicrobial therapy in the preceding four weeks were specifically requested. Volunteers were asked to return the stool sample and completed questionnaire by post to the Medical Microbiology Department in Liverpool. All samples were collected and received between August and September 2009.

Faecal samples were assessed according to the Bristol Stool Chart [22] and cultured for *C. difficile* by both direct and enrichment culture methods. Culture was performed with the faecal sample, either directly or after alcohol-shock treatment, on Brazier's cefoxitin-cycloserine egg yolk agar (CCEY, E & O Laboratories Ltd, Bonnybridge, UK) and then incubated for 48 h at 37°C in an anaerobic chamber. Similarly enrichment culture was performed by inoculating the faecal samples into the enrichment medium processed with and without alcohol-shock treatment [23]. Cooked meat broth (Oxoid Ltd, Basingstoke, UK) supplemented (after autoclaving) with sodium taurocholate (0.05%, Alfa Aesar Ltd, Heysham, UK), lysozyme (5 mg/L, Sigma-Aldrich, Poole, UK), cycloserine (500 mg/L, Bioconnections, Wetherby, UK) and cefoxitin (16 mg/L, Bioconnections) was used as enrichment medium. All positive samples were tested using a second aliquot from the same submitted specimen to confirm initial results and multiple colonies selected for PCR-ribotyping. A faecal sample known to be culture and toxin positive for *C. difficile* (ToxA+, ToxB+) was used as a positive control and a broth inoculated with *Escherichia coli* ATCC 25922 was used as a negative control with each batch of specimens cultured. Samples in enrichment broth were incubated for 48 h at 37°C and then sub-cultured on to Brazier's CCEY agar and incubated anaerobically for 48 h at 37°C. Potential *C. difficile* isolates were identified by their characteristic smell and colonial morphology, fluorescence under long wave UV light and a latex agglutination test for *C. difficile* somatic antigen (Oxoid Ltd, Basingstoke, UK). Isolates were stored on PROTECT beads (Technical Services Consultants Ltd, Heywood, UK) at −70°C.

Faecal samples were tested for the presence of both *C.difficile* glutamate dehydrogenase (GDH) and toxins A and/or B using C. DIFF CHEK-60 and TOX A/B II ELISA immunoassay kits (Techlab, Blacksburg, USA), respectively. Briefly, approximate 100 µg of faeces was mixed with 400 µl of the provided diluent. The content was then vigorously shaken for 1 minute and centrifuged at 10,000 g for 1 minute. Supernatants were then collected and the immunoassay test carried out following the manufacturer's instructions. Plates were read at 450 nm and corrected at 540 nm wavelength.

Stored isolates were sub-cultured for toxin detection and PCR-ribotyping at a later date. A multiplex PCR assay targeting a species-specific internal fragment of the triose phosphate isomerase (*tpi*) housekeeping gene, internal core sequences of both toxins A (*tcdA*) and B (*tcdB*) genes was used to confirm all isolates as *C. difficile* and verify their individual toxigenicity [24]. In addition, all isolates were tested for toxin expression. The isolate was inoculated into a 15 ml Brain Heart Infusion broth and cooked meat medium (Southern Group Laboratory Ltd, Corby, UK) and incubated anaerobically for 18–24 h at 37°C. Cultures were then centrifuged and the filtered supernatant used to test for the presence of toxins A and/or B using a TOX A/B II ELISA kit (Techlab, Blacksburg, USA). Isolates were typed by PCR-ribotyping according to the method described by Bidet *et al* [25] and compared to a panel of the commonest ribotypes circulating in the UK [26].

## Results

Faecal samples and questionnaires were received from 149 of the 206 volunteers approached; 23 declined consent while 34 did not respond. A summary of demographic information and faecal culture results is given in Table 1. All individuals in our study were aged over 70 years (median 81, IQR range, 72–90). The majority were female (79.9%), which reflects the higher enrolment rates by female volunteers as well as the presence of significant selective dropout since these volunteers are part of a large longitudinal cohort that has been monitored for more than 25 years [21].

**Table 1:** Demographic details of the studied subjects and summary of the *C.difficile* culture results.

PARAMETERS	RESULTS	N
**Age in complete years – Median (IQR)**	81 (72–90)	149
**Gender – Male vs. Female, n (%)**	30 (20.1%) vs. 119 (79.9%)	149
**CDI** * **history in the past 12 months – n (%)**	1 (0.7%)	145
**Gastro-Intestinal conditions – n (%)**	7 (4.7%)	149
**Any medications at baseline – n (%)**	137 (91.9%)	149
**Antibiotics in the last 4 weeks – n (%)**	16 (10.8%)	148
**PPIs** ** **or anti-acids at baseline – n (%)**	31 (21.4%)	145
**Carriage of ** ***Clostridium difficile*** ** – n (%)**	6 (4%)	149

*CDI *Clostridium difficile* infection.

**PPIs Proton pump inhibitors.

Among the individuals recruited, only one reported having CDI in the previous 12 months; however this individual was not culture positive for *C. difficile*. Nine (6%) volunteers had a history of major gastrointestinal conditions, such as Crohn's disease, ulcerative colitis, colectomy/cecotomy and colostomy. At the time of assessment, 137 of our volunteers (91.9%) were taking medication. Thirty-one (21.4%) were taking acid suppressive agents, but only 16 (10.8%) had taken any form of antimicrobial in the four weeks before providing a sample [Table 1].

All 149 stool samples submitted were considered formed and none were liquid (type 6 or 7 according to the Bristol stool chart). One sample was positive for *C. difficile* by both direct culture and enrichment culture. Another sample was negative by direct culture but positive by enrichment culture of the faecal sample treated with and without alcohol-shock. A further four samples were positive only by the enrichment culture method without alcohol-shock treatment [Table 2]. Three isolates were toxigenic (ToxA+, ToxB+) and four were non-toxigenic (ToxA-, ToxB-) based on our *in vitro* assay. No faecal samples were directly positive for Toxin A and/or B by the ELISA test. Only one of the 149 faecal samples tested was positive by GDH ELISA; this sample was also positive by direct culture. The other five samples that were positive by the enrichment culture method were negative for GDH. Three of these 6 individuals (50%) with *C. difficile* carriage were taking acid-suppressive agents compared to 28/139 (20.1%) of those who were free of *C. difficile* (RR = 2.48 [95% CI 0.89–4.70], X^2^ = 1.53, p = 0.22). Conversely, none of these positive subjects had history of CDI in the past 12 months, and neither did they report use of any antimicrobials in the previous 3 months prior to the recruitment.

**Table 2:** Summary of *C. difficile* strains isolated in the study.

	No Alcohol Shock		Alcohol Shock		Multiplex PCR					
Sample ID	Direct Culture	Enrichment Culture	Direct Culture	Enrichment Culture	TPI#	Toxin A	Toxin B	Toxigenic culture	GDH§ ELISA	PCR Ribotype
Sample 1	**−**	**+**	**−**	**+**	**+**	**+**	**+**	**+**	**−**	005
Sample 2	**−**	**+**	**−**	**−**	**+**	**+**	**+**	**+**	**−**	106
Sample 3*	**+**	**+**	**+**	**+**	**+**	**+/−**	**+/−**	**+/−**	**+**	003/039
Sample 4	**−**	**+**	**−**	**−**	**+**	**−**	**−**	**−**	**−**	009
Sample 5	**−**	**+**	**−**	**−**	**+**	**−**	**−**	**−**	**−**	026
Sample 6	**−**	**+**	**−**	**−**	**+**	**−**	**−**	**−**	**−**	026

# Triosephosphate isomerase.

§ Glutamate Dehydrogenase.

*Sample 3 consistently produced two distinct strains, one toxigenic (ToxA+/ToxB +) and the other non-toxigenic (ToxA−/ToxB−).

Four out of the seven strains identified in the volunteers were of non-toxigenic types based on toxin gene screening. PCR-ribotyping confirmed that the four non-toxigenic isolates had the following PCR-ribotypes: 009, 026 (n = 2) and 039. Two of them shared the same ribotype and one volunteer was found to concurrently carry two distinct strains: PCR-ribotypes 003 and 039 [Supporting Information - Figure S2], the former being toxigenic. This sample was positive on direct culture and was the single GDH positive faeces. Further samples were obtained from this volunteer and showed continued carriage of the strain type (PCR-ribotype 003) 12 months after the initial sample. No antibiotics or proton pump inhibitors were used during the one year follow-up. The other two toxigenic *C. difficile* strains observed in our cohort were PCR-ribotypes 005 and 106, which are amongst the most common strains circulating in the UK [26]. On further questioning it emerged that the individual carrying PCR-ribotype 005 worked regularly as a volunteer in a hospital, where this strain was prevalent.

## Discussion

The strength of this study is that it evaluates the presence of *C. difficile* in healthy elderly individuals living in the community, from a very well documented cohort [21]. All individuals were living in their own homes rather than in a nursing home care facility or in hospitals and we did not find evidence for geographical location clustering of positive volunteers. The overall carriage rate of 6/149 (4%) was considerably lower than that of a previous report by Brazier *et al.* [17], who found rates of 10.2% for carriage (47/284) and 6.3% (18/284) for eventual nosocomial acquisition amongst elderly patients admitted to 6 Welsh hospitals in 1999. In addition, two studies on healthy adults in Japan by Kato *et al.* in 2001 [14] and Ozaki *et al.* in 2004 [15] reported the presence of *C. difficile* in 94 of 1,234 subjects (7.6%) and 8 of 139 subjects (5.8%), respectively. The reported carriage rate with toxigenic strains were 4% in the two studies, compared with 2% in our study. An investigation conducted in long-term care facilities in the USA by Walker [12] found carriage rates of 7.1% in 225 subjects, whereas another American study [13] reported a rate of 5% (2 in 42 subjects) and a recent Irish investigation observed a prevalence of asymptomatic *C. difficile* carriage of 10 in 100 patients (10%), 7 of which were toxin positive (7%) [20]. These rates are considerably lower than the carriage rate of 19% reported by Simor *et al* [16] in nursing home patients in Canada. It is important to note that, with exception of the latter study [16], all the above investigations have used standard direct culture methods only and their figures would have been higher had they used enrichment culture methods. The fact we have also identified a sample which produced two distinct strains also highlights the need for testing multiple colonies isolated from the same specimen in order to further validate results.

While the Toxin A/B Elisa kit did not identify any positive samples, only one of the 149 faecal samples tested was positive by the GDH ELISA test. This sample was also positive by direct culture and remained culture positive 12 months after the first sample collection. The other five samples that were positive by enrichment culture only were negative for GDH. Although GDH assays reportedly have high specificity to *C.difficile* [27], it is very likely that the levels of GDH antigen in these samples were below the lower detection threshold for this test. Our results suggest that the GDH assay is significantly less sensitive than enrichment culture methods and reinforces the importance of enrichment culture for the screening of solid or semi-solid stools from healthy volunteers.

We found a range of distinct PCR-ribotypes consistent with the concept that the population of *C. difficile* in the community is significantly more diverse than in LCFs. The fact that two of the most common toxigenic PCR-ribotypes in the UK (i.e. 005 and 106) were found in our elderly cohort is not surprising. A previous screening study in Welsh hospitals found that over 50% of the strains isolated from elderly patients on admission were of PCR-ribotype 001, which was the most common strain circulating in hospital settings in the UK at the time [17]. Conversely, the endemic strain *smz*, the most frequent type isolated from CDI patients at that time and known to have caused multiple outbreaks in Japan, was not found in any healthy carriers by Kato *et al.* [14]. The epidemic strain type 027/NAP1/BI, which has largely accounted for the recent upsurge in the number of CDI cases, and has predominated in hospital settings in the United Kingdom [26] and Europe was not detected in our community-based elderly population. Although it is widely acknowledged that the epidemic PCR-ribotype 027 possesses additional virulence factors, such as hyperproduction of toxins [2] and increased sporulation rate [28], [29], it remains unclear whether its carriage is less likely to be asymptomatic in the elderly. Riggs *et al.* proposed that asymptomatic carriers are a potential source for transmission of epidemic and non-epidemic strains among LCF residents [9]. While this is likely to happen in a shared environment, it is uncertain whether carriage by healthy adults in the community is a potential source for community-associated CDI, particularly infections accounted by hypervirulent epidemic strain types, such as PCR-ribotype 027.

It is widely acknowledged that the diversity of *C. difficile* population is favoured by the absence of strong selective pressure, which is a process commonly triggered by antimicrobial agents. None of the six culture positive individuals gave a history of antimicrobial treatment in the past four weeks or hospital admission in the preceding 12 months. Three of the six positive individuals were receiving acid-suppressive agents. Although this trend is consistent with the suggestion that these agents facilitate colonisation by *C. difficile* [30]–[32], our study was underpowered to demonstrate any statistically significant differences given the low frequency of culture positive samples observed in our cohort.

The frequency and distribution of gastrointestinal carriage of *C.difficile* varies across different population groups. Indeed, Kato *et al.* found that carriage rates varied across occupational groups, such as university students, hospital workers, employees of a company and defence force personnel [14]. Age is another important predisposing factor. Asymptomatic carriage is higher in neonates and young infants than in adults, while at the same time, the distribution of symptomatic CDI cases is much more skewed towards the elderly. This suggests the existence of biological factors mediating initial colonisation and onset of the disease and highlights the importance of investigating both the symptomatic and asymptomatic carriage of *C. difficile* in the elderly. This study confirms that the carriage ratio of *C. difficile* amongst elderly individuals residing in their own homes in different geographical areas is likely to be lower than rates reported for individuals in LCFs, even after adopting enrichment methods to enhance the recovery of the organism. Hence, higher rates in LCFs probably reflect the greater probability of cross-contamination of the organism in these settings, as well as other contributing factors such as frailty, polypharmacy and increased use of medicines, such as proton pump inhibitors and antimicrobials.

Elderly individuals are a difficult group to recruit and one limitation of our study is the lack of follow-up to distinguish transient carriage from longer term colonisation. In a single individual we were able to conduct follow-up and demonstrated persistent carriage for one year. Another potential source of bias is the considerably higher rate of females in our cohort. This can partly be attributed to their greater longevity and to the lower enrolment and higher dropout rates among males of the original longitudinal study. The investigation of asymptomatic carriers in the community is important, not only to assess the host susceptibility to CDI, but also for the identification of novel pathogen factors associated with virulence and antimicrobial resistance. A clearer understanding is needed of why in some populations, such as neonates and children, *C. difficile* acts as a commensal but in others it becomes a pathogen. There is growing evidence that primary asymptomatic carriage of the organism may constitute a protective status against subsequent CDI by allowing the organism to mount an effective immune response. This, for example, constitutes the basis of recent vaccine trials for preventing recurrent CDI using multiple doses of non-toxigenic *C.difficile* strains as inducers [33]. A prospective investigation of a cohort of symptom-free *C. difficile* carriers to ascertain who might develop symptomatic infection could also help address this question.

## Supporting Information

Figure S1 **Geographic distribution of volunteers recruited by this study.** A. British Isles; B. Close-up in the North of England.
(TIF)

Figure S2 **PCR-Ribotyping results of the 7 strains recovered by both direct and enrichment methods.** Note that sample 3 generated isolates of two distinct ribotypes. “No-Alcohol” indicates no alcohol-shock treatment prior to the culture; “Alcohol” indicates alcohol-shock treatment prior to the culture “Enrichment” denotes enrichment culture, “Direct” denotes direct culture.
(TIF)
