# Supplementary figures and images for: Characterisation and Carriage Ratio of Clostridium difficile Strains Isolated from a Community-Dwelling Elderly Population in the United Kingdom
Source: PLoS One. 2011 Aug 23;6(8):e22804. doi: 10.1371/journal.pone.0022804 (PMC3160286; doi:10.1371/journal.pone.0022804)

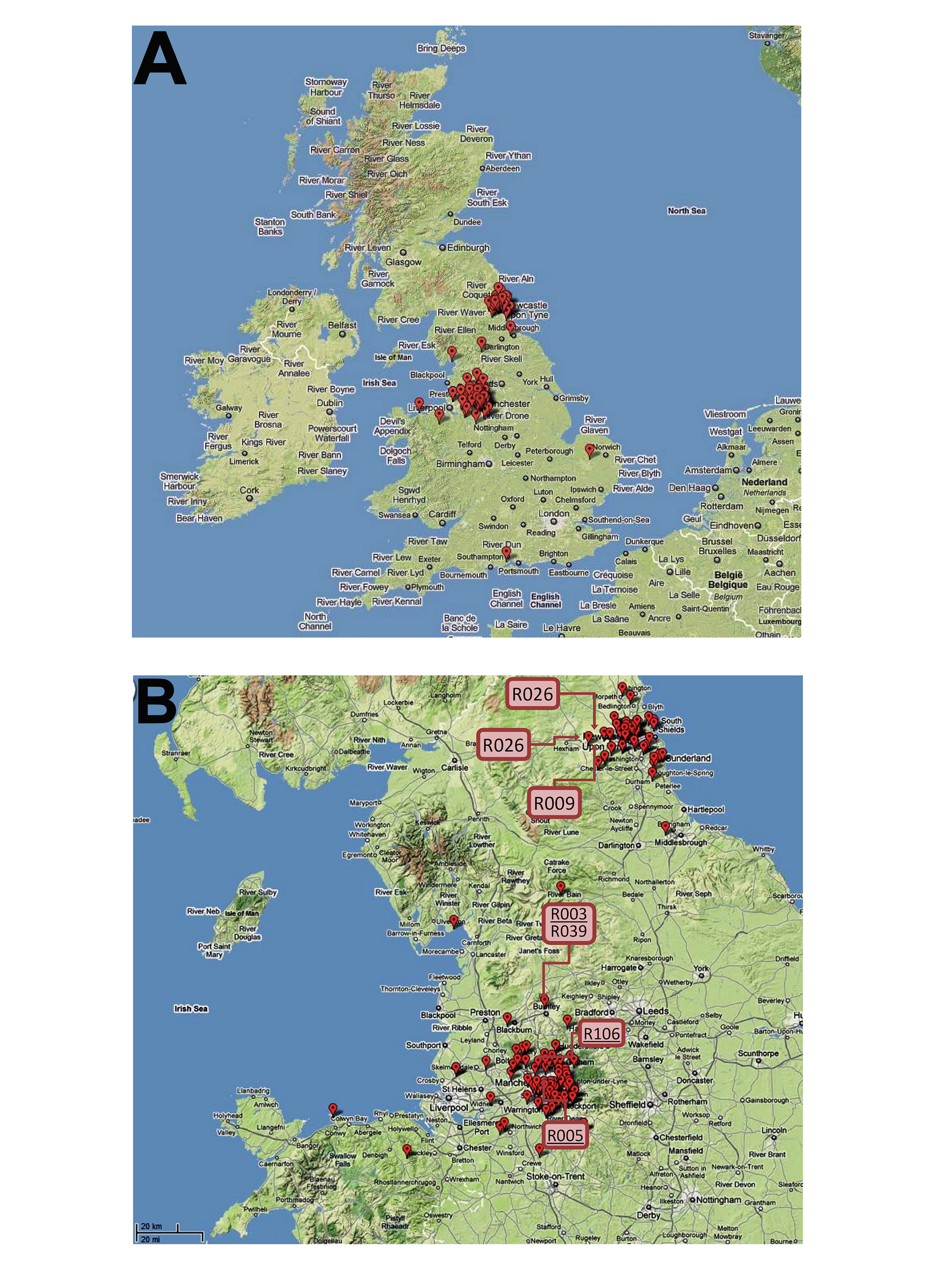

Supplement: Figure S1 — Geographic distribution of volunteers recruited by this study. A. British Isles; B. Close-up in the North of England. (TIF) [file pone.0022804.s001.tif]

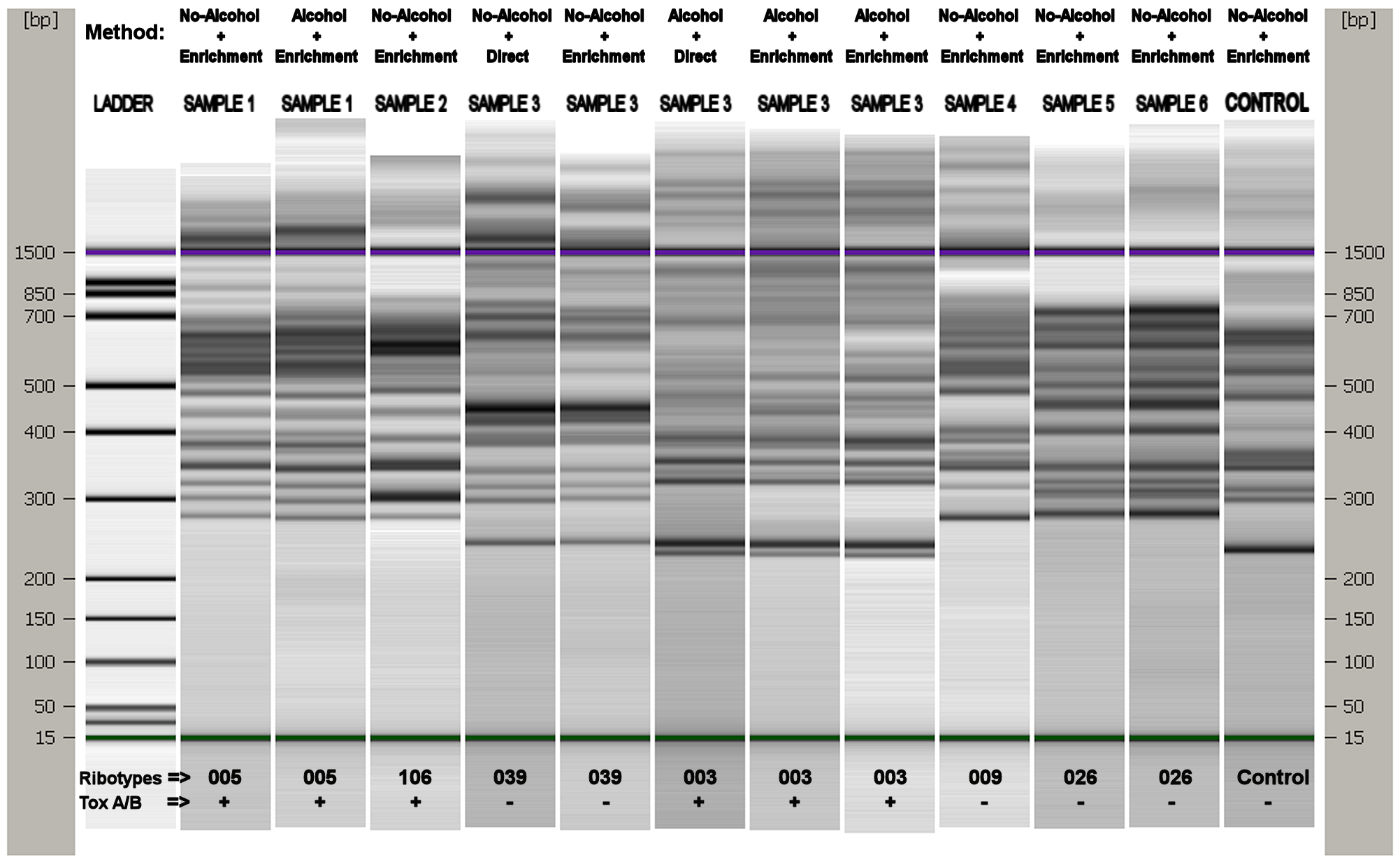

Supplement: Figure S2 — PCR-Ribotyping results of the 7 strains recovered by both direct and enrichment methods. Note that sample 3 generated isolates of two distinct ribotypes. “No-Alcohol” indicates no alcohol-shock treatment prior to the culture; “Alcohol” indicates alcohol-shock treatment prior to the culture “Enrichment” denotes enrichment culture, “Direct” denotes direct culture. (TIF) [file pone.0022804.s002.tif]
